# The Story of the Insane from Year to Year

**Published:** 1896-06-06

**Authors:** 


### THE STORY OF THE INSANE FROM YEAR TO YEAR.

Ninth Series.

### GLASGOW ROYAL ASYLUM.

The numbers on the first day of January were 495, and by the end of the year there were only 448 in residence. This decrease was caused by the transfer of pauper patients to the Lanark District Asylum, and now only 65 pauper patients remain in the institution. The admissions numbered 143; the recoveries, 52; cases discharged relieved, 111; and deaths, 27. The recoveries stand at 36·3 per cent. on the admissions, and the deaths at 4·2 on the average number resident. Of the admissions, 14 were hereditary, and 22 owing to drink. Ten of the deaths were due to cerebral diseases, and 4 to consumption. When referring to the reduction in the numbers consequent on the removal of the parish patients, the managers say the vacancies so caused will enable the asylum to provide suitable accommodation for the lower grades of private patients at rates of board within their means. And this is the true mission of all kindred institutions, although we fear it is an end often forgotten. It is much to be desired that in future reports a table should be inserted showing the number of cases received at low rates. The Commissioners say that "all parts of the institution were in excellent order."

